# Evaluation of Benzaldehyde as an Antibiotic Modulator and Its Toxic Effect against *Drosophila melanogaster*

**DOI:** 10.3390/molecules26185570

**Published:** 2021-09-13

**Authors:** Luiz Jardelino de Lacerda Neto, Andreza Guedes Barbosa Ramos, Thiago Sampaio de Freitas, Cristina Rodrigues dos Santos Barbosa, Dárcio Luiz de Sousa Júnior, Abolghasem Siyadatpanah, Morteza Nejat, Polrat Wilairatana, Henrique Douglas Melo Coutinho, Francisco Assis Bezerra da Cunha

**Affiliations:** 1 Laboratory of Semi-Arid Bioprospecting (LABSEMA), Regional University of Cariri, Crato 63105-000, CE, Brazil; luizjardelino@gmail.com (L.J.d.L.N.); andrezaurca@gmail.com (A.G.B.R.); darciolsjr@gmail.com (D.L.d.S.J.); cunha.urca@gmail.com (F.A.B.d.C.); 2 Graduate Program in Biological Chemistry, Regional University of Cariri, Crato 63105-000, CE, Brazil; thiagocrato@hotmail.com (T.S.d.F.); cristinase75@gmail.com (C.R.d.S.B.); 3 Laboratory of Microbiology and Molecular Biology (LMBM), Regional University of Cariri (URCA), Crato 63105-000, CE, Brazil; 4 Ferdows School of Paramedical and Health, Birjand University of Medical Sciences, Birjand 9717434765, Iran; 5 Master of Internal Surgery Nursing, Birjand University of Medical Sciences, Birjand 9717434765, Iran; nejatnursing@yahoo.com; 6 Department of Clinical Tropical Medicine, Faculty of Tropical Medicine, Mahidol University, Bangkok 10400, Thailand

**Keywords:** antibacterial, toxicity, benzaldehyde, resistance, insecticide

## Abstract

Products of natural origin remain important in the discovery of new bioactive molecules and are less damaging to the environment. Benzaldehyde is a product of the metabolism of plants, and similarly to oxygenated terpenes, it can have antibacterial activity against *Staphylococcus aureus* and toxic action against *Drosophila melanogaster*; we aimed to verify these activities. The broth microdilution tests determined the minimum inhibitory concentration (MIC) of benzaldehyde alone and in association with antibiotics and ethidium bromide (EtBr). Toxicity against *Drosophila melanogaster* was determined by fumigation tests that measured lethality and damage to the locomotor system. The results indicated that there was an association of norfloxacin and ciprofloxacin with benzaldehyde, from 64 μg/mL to 32 μg/mL of ciprofloxacin in the strain K6028 and from 256 μg/mL to 128 μg/mL of norfloxacin in the strain 1199B; however, the associations were not able to interfere with the functioning of the tested efflux pumps. In addition, benzaldehyde had a toxic effect on flies. Thus, the results proved the ability of benzaldehyde to modulate quinolone antibiotics and its toxic effects on fruit flies, thus enabling further studies in this area.

## 1. Introduction

The search for molecules with biological activity and toxicological safety regularly resort to natural products of plant, animal, and mineral origins to reduce costs, speed up the process of discovering new drugs, and discover new applications for already established drugs. Therefore, the search for products of natural origins that are biodegradable, toxicologically safe, and have insecticidal, bactericidal, or modulating activity has motivated several studies [1,2,3,4,5,6,7,8,9,10,11,12].

*Drosophila melanogaster*, known as the fruit fly, has proved to be an excellent model for its low maintenance cost, ease of handling, and for being outside the breeding stock of animals that require authorization for testing. It is also used as a model in studies of environmental contamination, toxicological responses, and drug and pesticide discovery [13].

The use of synthetic substances to combat insects has revealed at least two problems: one is environmental pollution when the substances are released into the environment, since many of them do not have metabolic routes for their transformation and elimination, the second is the resistance developed by insects to these compounds [10,14]. Toxicity tests and mobility assessment (negative geotaxis) produce relevant information for the selection of substances with potential insecticidal action against fruit flies [15,16,17,18,19,20].

Another type of resistance that concerns mankind is the bacterial resistance to the action of antibiotics. Bacteria, relatively quickly, can acquire and/or express genetic and phenotypic resistance, which is the result of complex interactions between genetic material; carriers of these materials; bacteria and their hosts; and the intense pressures promoted by man in an attempt of control and eradicate infections caused by bacteria [21]. Resistance to antibiotics occurs through various mechanisms such as (a) degradation of the antibiotic, (b) modification of the antibiotic target, (c) alteration of permeability, (d) ribosomal protection, (e) biofilm production, and (f) by the action of efflux pumps [22].

Interference in the operation of efflux pumps is one of the strategies used to discover compounds capable of returning the efficiency of antibiotics, whether their capacity to control and/or eliminate bacteria was lost or decreased. This ability can be verified by tests of association of the substance with ethidium bromide, which is extruded from bacterial cells by the action of efflux pumps. These compounds, even of natural origin, are called pump inhibitors, and they allow antibiotics to remain inside the bacterial cells for long enough to exert their effects on bacterial control [23,24].

Efflux pumps are subdivided into the following families: *ATP-binding cassette* (ABC) superfamily; *resistance-nodulation division* (RND) superfamily; *major facilitator* (MFS) superfamily; *multidrug and toxic compound extrusion* (MATE) family; *small multidrug resistance* (SMR) family; *proteobacterial antimicrobial compound efflux* (PACE) family, and *p-aminobenzoyl-glutamate transporter* (AbgT) family [25,26]. Each family has unique characteristics, such as what type of bacteria can express them and what substances are pumped out. These pumps are one of the factors that give bacteria resistance against existing antibiotics, and that resistance can lead to a “post-antibiotic era”, as described by the Centers for Infectious Diseases, in which more people will die from bacterial infection than from cancer [27,28].

Benzaldehyde is a natural compound biosynthesized by vegetables through the transcyclization of acetyl-CoA, an alcohol that is later oxidized to aldehyde, and it is a constituent of essential oils such as those found in *Cerasus subhirtella* and *C. serrulata*, accounting for 30% of the composition of these two plants [29,30].

Benzaldehyde is considered an environmentally safe compound because it is biodegradable. It has bactericidal activity against some strains with minimum inhibitory concentration (MIC) values ranging from 6 mM to 10 mM and against fungi this variation in MIC ranges from 8 mM to 10 mM; its insecticidal activity against *Galleria, mellonella*, and *Sitophilus oryzae* has also been reported [31,32].

The objectives of this work were to verify the antibacterial activity of benzaldehyde, confirm its ability to reduce the MIC of the standard antibiotics evaluated and EtBr, determine the interference of benzaldehyde on the mechanisms of the efflux pumps in *Staphylococcus aureus*, and determine the compound’s toxicity against *Drosophila melanogaster*.

## 2. Results

### 2.1. MIC Tests

Benzaldehyde had a MIC ≥ 1024 μg/mL. This value demonstrates that this substance has no relevant antibacterial activity against any of the *Staphylococcus aureus* strains tested. Antibiotics had MICs that ranged from 64 μg/mL to ≥1024 μg/mL. The antibiotics that showed the best antibacterial activity are shown in Table 1. The antibiotic erythromycin had an MIC of ≥1024 μg/mL against the RN4220 strain QacC pump, ciprofloxacin had an MIC of 64 μg/mL against the K2068 strain MepA pump; tetracycline had an MIC of 128 μg/mL against the IS-58 TetK strain TetK pump; oxacillin had an MIC of 162 μg/mL against the K 4100 strain MsrA pump; norfloxacin had an MIC of 256 μg/mL against 1199B strains NorA pump and 1199 (wild); and penicillin had an MIC of 362 μg/mL against the K 4414 QacA/B strain.

### 2.2. Antibiotic and Efflux Pump Inhibition Tests by Reducing the MIC of Ethidium Bromide

The following results demonstrate the MIC of the tested antibiotics, bromide, and the association of these with benzaldehyde in subinhibited concentrations with carbonyl cyanide m-chlorophenyl-hydrazone (CCCP) and chlorpromazine (the latter two are recognized as efflux pump inhibitors), thus confirming that the pump extrusion activity is active in the strains and can be inhibited by efflux pump inhibitors.

**Figure 1:**
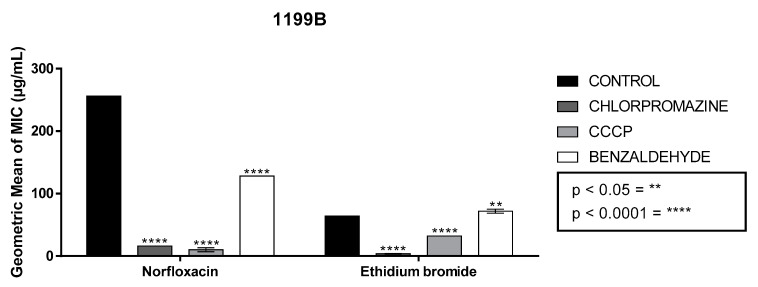
Ability of benzaldehyde to modulate the antibiotic action of norfloxacin and ethidium bromide in comparison with standard inhibitors (Strain 1199B). Legend: carbonyl cyanide m-chlorophenylhydrazone (CCCP). The values represent the geometric mean ± S.E.M. (standard error of the mean). Two-way ANOVA was used, followed by Bonferroni’s post hoc. ** = *p* < 0.05, **** = *p* < 0.0001.

In the tests carried out with the wild strain 1199, it was possible to observe that the association between norfloxacin and benzaldehyde in subinhibitory concentrations promoted a reduction in the MIC of the antibiotic (from 287 μg/mL to 256 μg/mL), and this same effect was observed in the test with ethidium bromide (from 80 μg/mL to 57 μg/mL). The assay with the 1199B strain, which expresses the NorA pump that provides resistance to fluoroquinolones, showed that benzaldehyde was not able to interfere with the operation of the pump in the same way that CCCP and chlorpromazine are able to, and this decreased the ethidium bromide MIC, in fact, an increase in MIC of ethidium bromide was observed, from 64 μg/mL to 72 μg/mL. However, the association of benzaldehyde with norfloxacin was able to reduce the MIC of norfloxacin, from 256 μg/mL to 128 μg/mL, revealing a potentiation of the bactericidal action (Figure 1).

When ethidium bromide was associated with benzaldehyde in the IS-58 strain, which expresses the TetK pump and acts by removing tetracyclines, the MIC was increased from 64 μg/mL to 256 μg/mL, indicating an increased resistance to tetracycline.

The MsrA pump responsible for the extrusion of macrolides is expressed in the RN4220 strain and was tested for the association of benzaldehyde with erythromycin and ethidium bromide; however, these associations did not show significant changes in the MIC of the antibiotic nor that of ethidium bromide.

**Figure 2:**
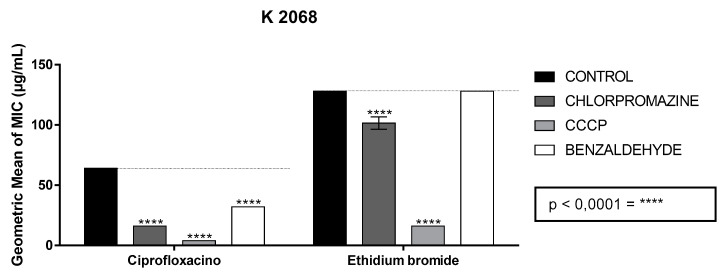
Ability of benzaldehyde to modulate the antibiotic action of ciprofloxacin and ethidium bromide in comparison with standard inhibitors (Strain K2068). Legend: carbonyl cyanide m-chlorophenylhydrazone (CCCP). The values represent the geometric mean ± S.E.M. (standard error of the mean). Two-way ANOVA was used, followed by Bonferroni’s post hoc. **** = *p* < 0.0001.

The association between benzaldehyde and ciprofloxacin revealed a decrease in the MIC of the antibiotic when tested against the K2068 strain, which expresses the MepA pump that acts on fluoroquinolones, especially on ciprofloxacin, reducing the MIC from 64 μg/mL to 32 μg/mL. Regarding the association with ethidium bromide, there were no changes in the MIC (Figure 2).

In the case of the K4414 strain, which expresses the QacA/B pump that expels beta-lactams, there was no change in the ethidium bromide MIC when associated with benzaldehyde. However, the penicillin MIC increased significantly when associated with benzaldehyde, from 362 μg/mL to 1024 μg/mL.

It was possible to observe that there was an increase in the MIC of ethidium bromide when associated with benzaldehyde (MIC/8), from 45 μg/mL to 64 μg/mL, when tested against the K4100 strain, which expresses the QacC pump that promotes the extraction of oxacillin.

### 2.3. Toxicity

#### 2.3.1. Mortality

**Figure 3:**
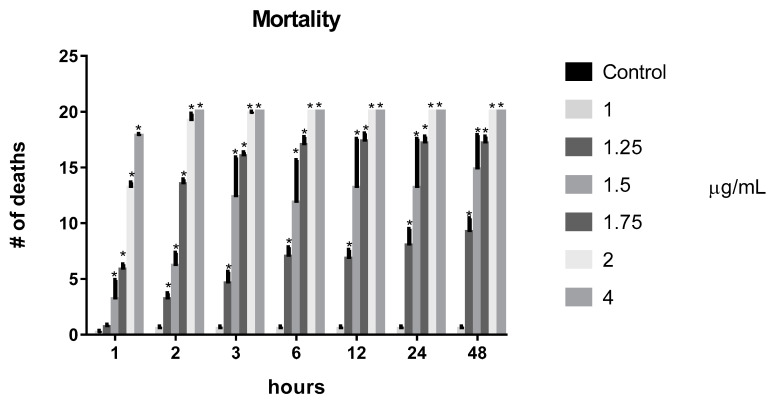
Effect of the benzaldehyde on *D. melanogaster* survival. Subtitles: Survival was analyzed at the indicated time points. Results are expressed as the Mean ± SD of the number of dead flies after each exposure time. * = *p* < 0.05 compared to the control.

Mortality is a toxic effect that can be observed after an organism’s exposure to certain compounds; obviously it depends on the concentration and time of exposure. The exposure of *D. melanogaster* to benzaldehyde by fumigation showed a mortality of 100% of the individuals within 2 h at a concentration of 4 µg/mL and all concentrations above 1.25 µg/mL showed a difference in mortality in relation to that of control (Figure 3).

#### 2.3.2. Damage to the Locomotor Apparatus

**Figure 4:**
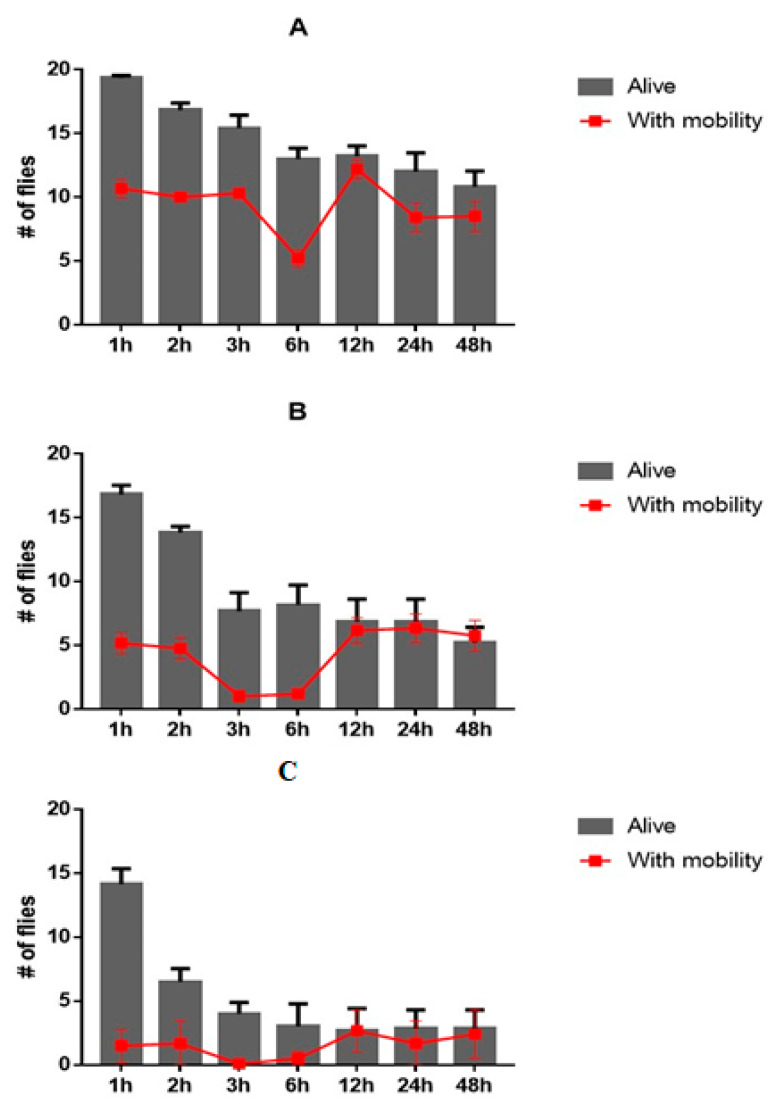
Relation between live flies and changes in mobility. The lines represent the number of flies that reached the top in the time intervals of exposure to benzaldehyde (**A** = 1.25 µg/mL, **B** = 1.5 µg/mL, and **C** = 1.75 µg/mL) and the histograms represent the number of live flies.

Damage to the locomotor system was measured by a negative geotaxis test. After exposure to a substance, the fly’s displacement capacity was verified. Those flies that exceeded 5 cm of vertical displacement within 5 s of the application of light mechanical shocks to the container were considered active. In Figure 4, we can see that in the first hour of reading it was possible to verify a difference in the number of flies that exceeded 5 cm of displacement in concentrations greater than 1 μg/mL.

From the mortality data, it was possible to perform a sigmoid regression to determine the average lethal concentration (LC50), which resulted in a value of 1.605 μg/mL (12.34 µg/L of air).

From the mortality and mobility tests, it was possible to establish a relationship between live flies and flies at the top (which exceed the vertical displacement of 5 cm) at concentrations of 1.25, 1.5, and 1.75 μg/mL. It is evident in the three figures that there is a simultaneous reduction in the number of live flies that manage to reach the top, up to the 6 h reading. After 6 h, it is possible to see an increase in the number of flies that reach the top and a relative stability in the number of deaths (Figure 4).

## 3. Discussion

Benzaldehyde is widely used in the food, beverage, and fragrance industries, and 7.1 thousand tons of this product are consumed annually by these industries as flavoring [33]. Several essential oils have benzaldehyde as a major constituent, making these products an important source of this substance. In Brazil some example are *Piptadenia viridiflora* oils, *Hyptis suaveolens*, and *Astronium* *graveolen* [34].

Benzaldehyde had an MIC greater than or equal to 1024 μg/mL for all strains tested, showing that it has no antibacterial activities at these concentrations for the tested strains of *Staphylococcus aureus*; however, other studies report antibacterial activity of benzaldehyde against *Bacillus anthracis* (8.0 mM or 850 μg/mL), *Pantoea conspicua* (10.0 mM—1060 μg/mL), and *Citrobacter youngae* (10.0 mM—1060 μg/mL). However, the activity observed in other bacteria can be explained by the fact that phenols and/or hydroxyl-benzaldehydes interact with the plasma membrane, causing its disintegration, and can also cause intracellular coagulation of bacterial cytosol; both mechanisms lead to cell death [32].

The association tests of benzaldehyde in subinhibitory concentrations (MIC/8) with antibiotics showed that there was a decrease in the MIC of norfloxacin (1199 strain and 1199B strain) and ciprofloxacin (K2068 strain), both fluoroquinolones that act by inhibiting enzymes involved in genetic processes (DNA gyrase and topoisomerase II). This fact may be the result of the action of benzaldehyde on the bacterial plasma membrane, promoting a change in permeability, as previously mentioned, thus increasing the availability of antibiotics inside the cell and resulting in a lower MIC for the antibiotic [35]. This justification is also based on the fact that resistance to quinolones is based on the action of efflux pumps, alteration of the antibiotic’s action site, and alteration of permeability. In the 1199 strain, this same decrease was observed when considering the association with ethidium bromide; however, an effect was not observed in the 1199B strain. These results reinforce the possibility of the permeability changing, since the 1199 strain does not have an efflux pump.

Studies show that polymers derived from benzaldehyde and from amines (4-hydroxybenzaldehyde and 2,4-dihydroxybenzaldehyde into amine-terminated polymers) have bactericidal activity, preventing bacterial growth, and that this capacity increases proportionally along with the number of presented phenolic hydroxyls [36]. Other studies show the relevance of the activity of benzaldehyde and oxygenated terpenes, such as thymol, nerol, dimethyl octanol, and estragol, in microbiological activities, demonstrating the importance of phenolic hydroxyls for this activity [37,38,39,40]. Vanilla, which is also an aromatic aldehyde, was able to modulate some antibiotics, decreasing the minimum inhibitory concentrations of norfloxacin, imipenem, gentamicin, and tetracycline and revealing an analogy between the modulation observed between benzaldehyde and quinolone antibiotics [41].

The inhibition of efflux pumps is a very important strategy so that a post-antibiotic era does not materialize. Efflux pump inhibitors can interfere with a pump’s operation in five ways: (1) by preventing energy flow in the pump, an example of this strategy is CCCP; (2) by decreasing the expression of the pumps, for example, by chlorpromazine [42,43], (3) by preventing the assembly of multi-component pumps, (4) by blocking the external channel of the pump, and (5) by generating a competitive inhibition with the antibiotic. Another strategy that should be considered is the chemical remodeling of antibiotics to the point that they are no longer substrates for the pumps, thus preventing their extrusion from the cell [44,45].

Association tests with ethidium bromide are important to elucidate the participation of pumps in the extrusion of substances, because ethidium bromide is eliminated by these pumps [39,46]. A compound that has the capacity to inhibit efflux pumps may be acting in any one of the five previously mentioned ways. However, in none of the strains that were tested was benzaldehyde able to decrease the MIC of ethidium bromide, and in the IS-58 and K4100 strains, there was an increase in the MIC of ethidium bromide. In the assay with the K4414 strain that expresses the QacA/B pump, the association of the antibiotic with a subinhibitory concentration of benzaldehyde increased the MIC, this result needs to be further investigated, but there is a possibility that a complexation occurred between penicillin and other substances that caused less availability in the culture medium of free and active penicillin molecules; this situation could be classified as a drug–drug interaction, and this hypothesis should be further investigated [47,48,49].

Benzaldehyde did not present itself as a possible efflux pump inhibitor, because it did not decrease the MICs of EtBr; however, the enhancement of the activity observed between it and antibiotics (ciprofloxacin and norfloxacin) may suggest the influence of resistance mechanisms other than efflux pumps, such as degradation of the antibiotic, modification of the antibiotic target, and alteration of permeability [22].

The toxicity assay against *Drosophila melanogaster* revealed an LC_50_ of 1.60 mg/mL of benzaldehyde, other studies have also reported the toxic potential of benzaldehyde against some insect species such as *Aedes aegypti*, *Culex quinquefasciatus* [50], *G. mellonella* [32], and *Sitophilus oryzae* [31].

Studies have already shown that several natural products have insecticidal potential. For example, benzaldehyde has activity against *Meloidogyne incognita*, which is a nematode that has a large impact on soybean cultivation by causing loss of productive crops, and against *Strongyloides ransomi*, a widely used model in the discovery of anthelmintic drugs [34,51,52]. The molecular similarity of benzaldehyde with oxygenated terpenes (all having an oxygen linked to an aromatic ring and similarities in physical properties) raises the hypothesis that the lethal damage is caused by increased oxidative stress and changes in the respiratory chain; future studies should focus on this hypothesis [53,54,55]. However, the target species must be considered, as benzaldehyde can be used as an attractant for bees, thus promoting pollination, as already reported by some studies [56,57].

The damage tests of the locomotor system indicated that benzaldehyde acts quickly, within about 1 h of exposure, and all groups with a concentration greater than 1 mg/mL showed a difference in locomotion when compared to that of the control. Again, this effect can be analogous to that produced by oxygenated terpenes, due to chemical similarity. Thus, in the future we should investigate the participation of enzymes capable of reducing oxidative stress, such as glutathione S-transferase (GST), catalase (CAT), and superoxidodesmutase (SOD); target proteins involved in oxidative stress [54]; and the participation of the nervous system [58].

The interpolation of the results of mortality and damage to the locomotor system allowed us to verify that after six hours the mortality curve was stable while the curve that indicates the number of flies with minimal damage to the locomotor system showed growth. Superoxide dismutase and glutathione S-transferase can cause cells to express the protein Hsp70, a Chaperone 70 (~kDa) that demonstrates a late action in the protection against oxidative stress. This is one more point to be clarified in new studies of benzaldehyde [54].

## 4. Material and Methods

### 4.1. Culture Media

The following culture media were used to perform microbiological tests: heart infusion agar (HIA) (Difco Laboratorises Ltd., Franklin Lakes, NJ, USA), prepared according to the manufacturer’s instructions; brain heart infusion (BHI) (Acumedia Manufacturers Inc., Lansing, MI, USA) prepared at a concentration of 10%; and glycerol culture media, prepared according to the manufacturer’s instructions.

### 4.2. Microorganisms

The following strains of *S. aureus* used were: 1199 wild strain; 1199B, carrying the norA Small D gene that encodes the NorA efflux protein, resistant to hydrophilic fluoroquinolones; IS-58, endowed with the plasmid PT181 and carrying the gene that expresses the efflux protein of TetK tetracycline; K2068 strain that has the MepA pump, decoded in the mepA gene that confers resistance to ciprofloxacin; K4100 strain that has the QacC pump, decoded by the qacC gene and conferring resistance to oxacillin; K4414 strain that expresses the QacA/B pump, decoded by the qacA/B gene, which promotes resistance against penicillin; and RN4220, carrying the pUL5054 plasmid, which carries the gene that encodes the macrolide efflux MsrA protein (Table 2). The strains were kindly provided by Prof. S. Gibbons (University of London) and Prof. Glenn Kaatz (Wayne State University). All strains were initially kept on blood agar to prove the type of strain (Laboratórios Difco Ltd., Franklin Lakes, NJ, USA) and then were transferred into one of two stocks: heart infusion agar (HIA, Difco) at 4 °C or glycerol in a freezer at 80 °C. The plasmid-bearing strain was maintained in culture medium under subinhibitory conditions of antibiotics, with the objective of showing expression of resistance genes and plasmids without loss of the strain.

### 4.3. Origin and Preparation of Substances

Benzaldehyde was purchased from SIGMA Chemical Co., St. Louis, USA and *carbonyl cyanide m-chlorophenyl-hydrazone* (CCCP) and clopromazine were purchased from SIGMA Chemical Co., St. Louis, USA and Aché, respectively. CPMZ and EtBr were dissolved in sterile distilled water, while CCCP was dissolved in methanol/water (1:3, *v*/*v*). The antibiotics and benzaldehyde were diluted in dimethyl sulfoxide (DMSO) and in sterile water. All substances were diluted to a standard concentration of 1024 μg/mL and the proportion of DMSO used was less than 5%. The antibiotics used were specific to the pumps from each bacterium: erythromycin for the MsrA pump expressed in the RN4220 strain; tetracycline for the TetK pump expressed in the IS-58 strain; norfloxacin for the NorA pump contained in the 1199B strain and for the 1199 wild strain; ciprofloxacin for the MepA pump expressed in the K2068 strain; penicillin for the QacA/B pump expressed by K4414; and oxacillin for the QacC pump expressed by the strain K4100.

### 4.4. Preparation and Standardization of the Inoculum

In the tests to obtain the MIC, inoculants were prepared from the stocks and were cultured again in a solid *heart infusion agar* medium and kept at 37 °C. From this solid medium, inoculants were made in test tubes containing sterile saline solution, and these inoculants were based on the Mcfarland 0.5 scale that corresponds to 1 × 10^8^ (CFU)/mL (colony forming units). This standard inoculum method was used both in the MIC tests of the substances and in the verification of the efflux pump inhibition.

### 4.5. MIC Tests

The MIC was determined for benzaldehyde according to the broth microdilution method proposed by Javadpour [59] with adaptations. The inoculants were prepared 24 h after sowing the strains. Subsequently, the microtubes were filled with 900 µL of BHI and 100 µL of the inoculum. The plates were then filled with 100 µL of the final solution. Microdilution was performed with 100 µL in serial dilutions up to the penultimate cavity (1:1), the last one being used as a growth control. The concentrations of the compounds ranged from 8.0 μg/mL to 512 μg/mL. The plates were incubated at 37 °C for 24 h and bacterial growth was evaluated with resazurin (7-hydroxy-10-oxidophenoxazin-10-ium-3-one) at a concentration of 400 μg/mL. Resazurin was oxidized in the presence of an acid medium caused by bacterial growth, causing the color to change from blue to pink [60]. MIC was defined as the lowest concentration at which no growth could be observed [61]. Microbiological tests were performed in triplicate.

### 4.6. Efflux Pump Inhibition Tests by Modifying the MIC of the Antibiotic and Ethidium Bromide

In order to observe whether benzaldehyde acts as a potential inhibitor of the efflux mechanism, a comparative study between the effects of the standard inhibitors of the efflux pump was used, evaluating the ability of both to decrease the MIC of antibiotics and EtBr (controls). PPE standard CCCP and CPMZ were used to prove the expression of the NorA pump by the strains tested. The inhibition of the efflux pump was tested using a subinhibitory concentration (MIC/8) of the inhibitors and of benzaldehyde. In the tests, 170 µL of each suspended bacterial inoculum in a saline solution, corresponding to the Mcfarland 0.5 scale, which corresponds to 1 × 10^8^ (CFU)/mL, was added to the inhibitors and benzaldehyde (MIC/8) and completed with BHI. These were then transferred to 96-well microdilution plates, to which 100 µL of antibiotic or EtBr were added in serial dilutions (1:1) ranging from 512 to 0.25 μg/mL. The plates were incubated at 37 °C for 24 h and bacterial growth was evaluated with resazurin (7-hydroxy-10-oxidophenoxazin-10-ium-3-one) at a concentration of 400 μg/mL. Resazurin was oxidized in the presence of an acid medium caused by bacterial growth, causing the color to change from blue to pink [60]. MIC was defined as the lowest concentration at which no growth could be observed [61]. The MIC of the controls was evaluated using only microdiluted plates with antibiotics and EtBr.

### 4.7. Toxicity Test

#### 4.7.1. Breeding and Stocking of *Drosophila melanogaster*

*Drosophila melanogaster* (Harwich strain) was obtained from the National Species Stock Center (Bowling Green, OH USA). The flies were bred according to the methodology already described [55], in 340 mL glass containers and with the medium containing 83% corn mass, 4% sugar, 4% lyophilized milk, 4% soy bran, 4% wheat bran, and 1% wheat salt. When cooking the mixture, 1 g of nipagin (methylparaben) was added. After cooling in the growth vessels, 1 mL of solution containing *Saccharomyces cerevisiae* was added. The flies were kept at a temperature of 26 °C and a relative humidity of 60%. All tests were performed with the same strain [55].

#### 4.7.2. Mortality Test

The tests were performed according to the method proposed by Cunha [55], with some modifications. Twenty adult flies (male and female) were placed in 130 mL glass containers (6 cm high and 6.5 cm in diameter), containing tracing paper at the bottom, previously prepared with 1 mL of 20% sucrose solution. Inside the container lid had filter paper that was impregnated with benzaldehyde in concentrations of 1 µg/mL (8.03 µg/L of air), 1.25 μg/mL (10.04 µg/L of air), 1.5 μg/mL (12.05 µg/L of air), 1.75 μg/mL (14.06 µg/L of air), 2 μg/mL (16.06 µg/L of air), and 4 μg/mL (32.12 µg/L of air). In the control group, only sucrose was added to the tracing paper. Throughout the entire procedure, a 12 h light/dark cycle was maintained, the temperature was controlled at 26 °C, and 60% relative humidity was maintained. The tests were performed in triplicate, where each “*n*” was composed of two containers. The readings for mortality (being determined by the number of deaths) were performed 1, 2, 3, 6, 9, 12, 24, and 48 h after applying the compound to the filter paper.

#### 4.7.3. Negative Geotaxis Test

The damage to the locomotor system was verified through the negative geotaxis test described by Coulom and Birman. Readings were taken of live flies 1, 2, 3, 6, 9, 12, 24, and 48 h after being exposed to benzaldehyde. The flies started at the bottom of the containers, and after 5 s, the number of flies that reached a 5 cm height in the container were counted. The tests were repeated twice at one-minute intervals. The results are presented as average time (±) SD obtained in two independent experiments [62].

### 4.8. Statistical Analysis

#### 4.8.1. Statistical Analysis of Microbiological Results

The test results were done in triplicate and expressed in geometric average. One-way ANOVA was used for statistical analysis, followed by Tukey or Bonferroni post hoc using GraphPad Prism 5.0 (GraphPad Software, San Diego, CA, USA). In some analyses, the *t* test was used.

#### 4.8.2. Statistical Analysis of Toxicity Results

The analysis of toxicity data was performed using a two-way ANOVA test, followed by a Tukey multiple comparison test. There was no statistical difference with the same concentration as a function of time.

## 5. Conclusions

Benzaldehyde presented, in association with quinolones, a reduction in MICs against resistant strains, demonstrating a potentiating antibiotic activity of the product for this group of antibiotics. There was no influence of benzaldehyde on any of the seven pumps in the tests that resulted in a decrease in MIC for ethidium bromide. However, it was possible to verify the toxicity of benzaldehyde against *Drosophila melanogaster*, clearly showing that benzaldehyde showed high toxicity in this eukaryotic model. Thus, this demonstrated toxicity against insects, and the modulation of quinolone antibiotics against *Staphylococcus aureus* should inspire the investigation of benzaldehyde’s potential as a modulator and bioinsecticide.

## Figures and Tables

**Table 1:** MIC of antibiotics and benzaldehyde against *Staphylococcus aureus*.

Strain	Efflux Pump	MIC of Antibiotics	MIC of Benzaldehyde	Association (Antibiotic + MIC/8 of Benzaldehyde)
1199	-	Norfloxacin 287 μg/mL	≥1024 μg/mL	256 μg/mL
1199B	NorA	Norfloxacin 256 μg/mL	≥1024 μg/mL	128 μg/mL
IS-58	TetK	Tetracycline 128 μg/mL	≥1024 μg/mL	128 μg/mL
K2068	MepA	Ciprofloxacin 64 μg/mL	≥1024 μg/mL	32 μg/mL
RN4220	QacC	Erythromycin ≥ 1024 μg/mL	≥1024 μg/mL	1024 μg/mL
K4414	QacA/B	Penicillin 362 μg/mL	≥1024 μg/mL	1024 μg/mL
K4100	MsrA	Oxacillin 162 μg/mL	≥1024 μg/mL	143 μg/mL

**Table 2:** Tested strains.

Strain	Pump	Family	Gene	Antibiotic
1199	-	-	-	Norfloxacin
1199B	NorA	MFS	*norA* Smal D	Norfloxacin
IS-58	TetK	MFS	Plasmid PT181	Tetracycline
K2068	MepA	MATE	*MepA*	Ciprofloxacin
K4100	QacC	SMR	*qacC*	Oxacilin
K4414	QacA/B	SMR	qacA/B	Penicillin
RN4220	MsrA	MRSA	*msr*A pUL5054	Erythromycin

## Data Availability

The data present in this study are available on request from the corresponding autor.

## Sample Availability

The samples in this study are available on request from the corresponding autor.
